# Effects of Habitat Types on Pollinators and Seasonal Variations in Plant–Insect Pollination Networks in Nanchong, China

**DOI:** 10.3390/insects17090930

**Published:** 2026-09-04

**Authors:** Qi Wang, Yunong Zou, Xiaokang Zhou, Jie Zhang, Xiushan Li

**Affiliations:** 1 Key Laboratory of Wildlife Conservation in Southwest China, Ministry of Education, College of Life Sciences, China West Normal University, Nanchong 637002, China; 2 Sericulture Research Institute, Sichuan Academy of Agricultural Sciences (Institute of Special Economic Animals and Plants, Sichuan Academy of Agricultural Sciences), Nanchong 637000, China; 3 Department of Animal Ecology and tropical Biology, Julius-Maximilians-University of Würzburg, Am Hubland, 97074 Würzburg, Germany

**Keywords:** land use pattern, habitat type, pollinator, pollination network dynamics, conservation strategy

## Abstract

Land-use type acts as a key anthropogenic factor shaping the diversity of pollinator communities. Different habitats indirectly alter pollinator species richness, abundance and community structure by regulating vegetation coverage and the abundance of host and nectar plants. To clarify the mechanisms underlying habitat effects on pollinator assemblages, we conducted transect surveys to investigate pollinator communities across three typical habitats (forest, cropland and urban area) in Nanchong City. Our results revealed that pollinator alpha diversity was significantly higher in forests than in croplands and urban areas, with urban habitats harboring the lowest species diversity. Vegetation coverage and nectar plant richness were identified as the primary environmental drivers of pollinator diversity. The plant–pollinator interaction network exhibited pronounced seasonal dynamics: spring networks displayed a distinct nested architecture dominated by generalized mutualistic interactions; networks shifted gradually from generalized to specialized links in early summer; and only a small number of specialist pollinators persisted in late summer to early autumn. Accordingly, retaining wild flowering herbs adjacent to pollinator-dependent crops and conserving autumn- and winter-blooming plant species can sustain continuous floral food resources for pollinators, promote pollinator richness, stabilize pollinator communities, and enhance the ecosystem service of pollination.

## Simple Summary

Habitat types alter pollinator species richness, abundance and community structure by regulating vegetation coverage and the abundance of host and nectar plants. To clarify the mechanisms underlying habitat effects on pollinator assemblages, a transect survey was conducted across three typical habitats (forest, cropland and urban area) in Nanchong City. The results revealed that pollinator alpha diversity was significantly higher in forests than in croplands and urban areas. Vegetation coverage and nectar plant richness were identified as the primary drivers of pollinator diversity. The plant–pollinator interaction networks exhibit distinct seasonal dynamics, shifting progressively from generalized to specialized structures throughout the year. Reducing pesticide application and developing pollinator-friendly agricultural systems are critical strategies for the conservation and sustainable management of pollinator communities. Furthermore, retaining wild autumn- and winter-blooming plant species can sustain pollinator populations, promote pollinator richness, and enhance regional pollination ecosystem services.

## 1. Introduction

Pollinators serve as irreplaceable core functional groups in terrestrial ecosystems and agricultural production, as they support the sexual reproduction of most wild flowering plants and cultivated crops, sustain stable food production and ecosystem function, and are closely associated with human well-being [1]. Insect pollination services deliver tremendous economic benefits worldwide, with insect-dependent crop production generating an annual global economic output of US$235 billion to US$577 billion in 2015 [2]. Statistically, pollinator-mediated pollination contributes to 5–8% of global agricultural food production [2], and this proportion rises to 8.45–12.88% in Southwestern China [3]. Beyond ecological and economic values, pollinators are highly sensitive to environmental changes and can be utilized as reliable bioindicators for ecological quality evaluation and long-term environmental monitoring, underscoring the crucial theoretical and practical significance of pollinator diversity research [4,5]. Nevertheless, global pollinator populations are facing widespread decline driven by multiple anthropogenic and environmental stressors, including land-use changes, intensive agricultural practices, climate change, and invasive alien species [1,6,7,8,9]. Such anthropogenic disturbances not only reduce plant fruit-setting efficiency, degrade ecosystem structural stability and biodiversity, and threaten global food security, but also lead to insufficient pollination supply, declined crop yields, and deteriorated agricultural product quality [1,2]. Among all driving factors, land-use change is identified as one of the dominant threats to pollinator communities [1,10,11]. In this context, exploring pollinator diversity dynamics across diverse habitats helps clarify the impacts of different land-use types on biodiversity. This scientific evidence can further guide the formulation and implementation of pollinator-friendly land-use management strategies, mitigate the adverse effects of human disturbances, promote the conservation and sustainable utilization of pollinators, improve pollination service efficiency, and ultimately enhance human well-being.

Human land-use disturbances affect ecosystems primarily through two pathways: the conversion of natural ecosystem types and the intensive anthropogenic management of natural landscapes [11]. Typical land conversion activities include deforestation and grassland reclamation for cropland expansion and crop production, as well as to obtain higher livestock yields; mining and tourism development in forested areas that degrade native vegetation and alter original ecological functions; and rapid urbanization that converts peri-urban agricultural land into urban construction land [12]. The intensive anthropogenic management of natural landscapes includes monoculture farming, extensive pesticide application, and grassland overgrazing [11,13,14]. In addition, ecological restoration practices such as the conversion of sloping farmland back to forested land and grassland also reshape regional land-use patterns in China [15,16,17].

In terms of ecosystem management, intensive agricultural practices, large-scale monoculture cropping, and widespread pesticide application substantially modify the structure of agroecosystems [11,13,14]. To increase timber yields, extensive natural old-growth forests are logged and replaced by monoculture plantations, which greatly simplify forest community structures and ecological complexity [18,19]. Meanwhile, long-term overgrazing for livestock production severely disrupts the stability and functioning of grassland ecosystems [15,20]. These land-use transformations and intensive management practices profoundly alter the structure, function, and stability of natural ecosystems, resulting in significant biodiversity degradation and posing severe threats to pollinator communities [21,22]. Such drastic land-use changes have been pervasive throughout the ecological management history of China. During the 1980s and 1990s, long-term excessive deforestation caused a temporary decline in forest coverage and simplified forest stand structures, which substantially impaired key ecosystem services including soil and water conservation and biodiversity conservation [22,23]. Synchronously, China’s agricultural systems have undergone a transition from traditional smallholder farming to intensive commercial cultivation. Grassland management has shifted from chronic overgrazing toward livestock carrying capacity regulation and ecological restoration [20]. Accelerated urban expansion has continuously encroached on peripheral agricultural land, leading to persistent restructuring of regional land-use patterns and profound impacts on native biodiversity and ecosystem sustainability [20].

Insects represent the most species-rich taxonomic group on Earth and are highly susceptible to habitat disturbances. Over the past decades, the abundance and species richness of global insects have declined sharply, triggering synchronous declines of biota across multiple trophic levels [24,25]. Since the 21st century, wild pollinators have been experiencing the sixth global biodiversity decline crisis [26]. Long-term monitoring data from the European Butterfly Monitoring Scheme demonstrate that intensive agroforestry, inadequate grassland management and habitat loss have jointly reduced the composite abundance index of butterfly pollinators by 39% since 1990. Among 435 assessed butterfly species, 37 are classified as threatened taxa on red lists [27]. Therefore, systematically disentangling the response mechanisms of pollinators to environmental changes carries vital practical implications for both ecological conservation and modern agricultural production.

Land use and its ecological consequences have remained a central research hotspot in global conservation ecology [28,29]. Cumulative studies have gradually uncovered the relationships between land-use types, environmental factors and pollinator assemblages. Combined land-use patterns and regional climate jointly shape pollinator biodiversity, while landscape heterogeneity substantially facilitates insect niche differentiation [29]. Nevertheless, sixty years of long-term monitoring data in Europe reveal that variations in butterfly community structure are primarily regulated by habitat transformation, with climate change exerting relatively weak independent driving effects [30,31]. This finding confirms that distinct habitat types impose differential filtering effects on pollinator communities. The conversion of tropical primary forests to farmlands drastically reduces pollinator diversity [7]. The adverse impacts of land-use change exhibit obvious taxonomic specificity, depending on pollinators’ biogeographic ranges, host plant specialization and breeding habitat preferences [32]. Higher land-use intensification weakens the resistance of regional pollination services to anthropogenic disturbances [33]. Different pollinator groups show divergent responses to habitat conditions and cropping regimes: bees and wasps prefer miscanthus habitats over rapeseed farmlands, and the abundance of all pollinators and nectar plants is consistently higher along field margins than within field interiors [34].

Woodlands with complex vegetation structure and high canopy coverage are high-quality refugia for conserving pollinator communities. Elevated vegetation coverage and floral diversity significantly boost pollinator species richness [34,35]. However, inconsistencies persist among existing research conclusions: increased floral cover suppresses insect diversity in croplands but exerts positive effects on pollinator assemblages in grasslands. Such disparities are attributed to differences in resource supply and microhabitat characteristics between the two land-use categories [36]. Notably, the negative ecological impacts induced by land-use conversion feature lag effects; the influences of historical anthropogenic disturbances do not vanish rapidly following habitat restoration, and can persistently affect pollinator communities over the long term. Meanwhile, heterogeneous mosaic landscapes composed of interspersed multiple habitat types deliver potential conservation benefits. Diverse habitats provide complementary foraging and nesting resources for pollinators, thereby maintaining the integrity of pollinator communities [37].

Habitat loss and degradation driven by land-use change not only alter the spatial distribution patterns of pollinators and host plants, but also profoundly restructure the overall architecture of interspecific mutualistic networks [38]. As a core analytical framework for quantitatively characterizing plant–insect mutualisms, pollination networks provide standardized approaches to explore pollination system diversity, seasonal temporal dynamics, functional structures and plant–pollinator coevolution [39]. A stable pollination network structure underpins the persistence of biological communities and ecosystem homeostasis. Over the past decade, qualitative and quantitative pollination network analysis have become mainstream methods for decoding pollinator decline mechanisms. They are widely applied to disentangle multiple drivers of global pollinator loss and offer theoretical support for formulating pollinator conservation strategies [40]. Pollination networks are constructed primarily based on floral visitation frequency, a metric that quantifies interaction strength between plants and pollinators and reflects regional plants’ reliance on insect pollination services.

Various anthropogenic disturbances drastically alter pollination network architecture. Artificial nocturnal urban lighting not only directly inhibits nocturnal pollinators and their mutualistic interactions, but also indirectly disturbs diurnal pollinator assemblages [41]. Low-intensity extensive land management preserves the diversity and generalized connections within plant–insect networks to sustain long-term community stability [42]. Sufficient resource redundancy in forest habitats buffers species extinction risks and prevents cascading secondary extinctions within networks [43]. Environmental filtering resulting from habitat isolation elevates the modularity of pollination networks, while generalist pollinators such as bees strengthen network connectivity and guarantee the structural integrity of entire networks [44].

In summary, systematically illustrating the differential effects of diverse land-use types on pollinator communities and revealing the intrinsic mechanisms through which anthropogenic land disturbances threaten pollinator assemblages bear great theoretical and practical significance. The findings can provide scientific foundations for regional biodiversity conservation and sustainable agroforestry management. In this study, we take multiple typical habitats across Nanchong City, Sichuan Province, as research subjects, systematically analyze the effects of habitat types on pollinator communities, and clarify monthly seasonal dynamics of plant–insect pollination networks. This research aims to supply theoretical references and practical guidance for the conservation and sustainable utilization of local wild pollinators.

Against this research background, this study proposes three core hypotheses regarding butterfly community variation between different habitats: (1) significant differences exist in pollinator community characteristics among different land-use types, with obvious divergence in species richness, abundance, community dominance, and Shannon–Wiener diversity index; (2) the decline of pollinator diversity is driven by the synergistic effects of vegetation characteristics and environmental factors; and (3) the plant–pollinator interaction networks in Nanchong City exhibit significant seasonal variation in stability, specialization, and modularity.

## 2. Materials and Methods

### 2.1. Study Area

**Figure 1:**
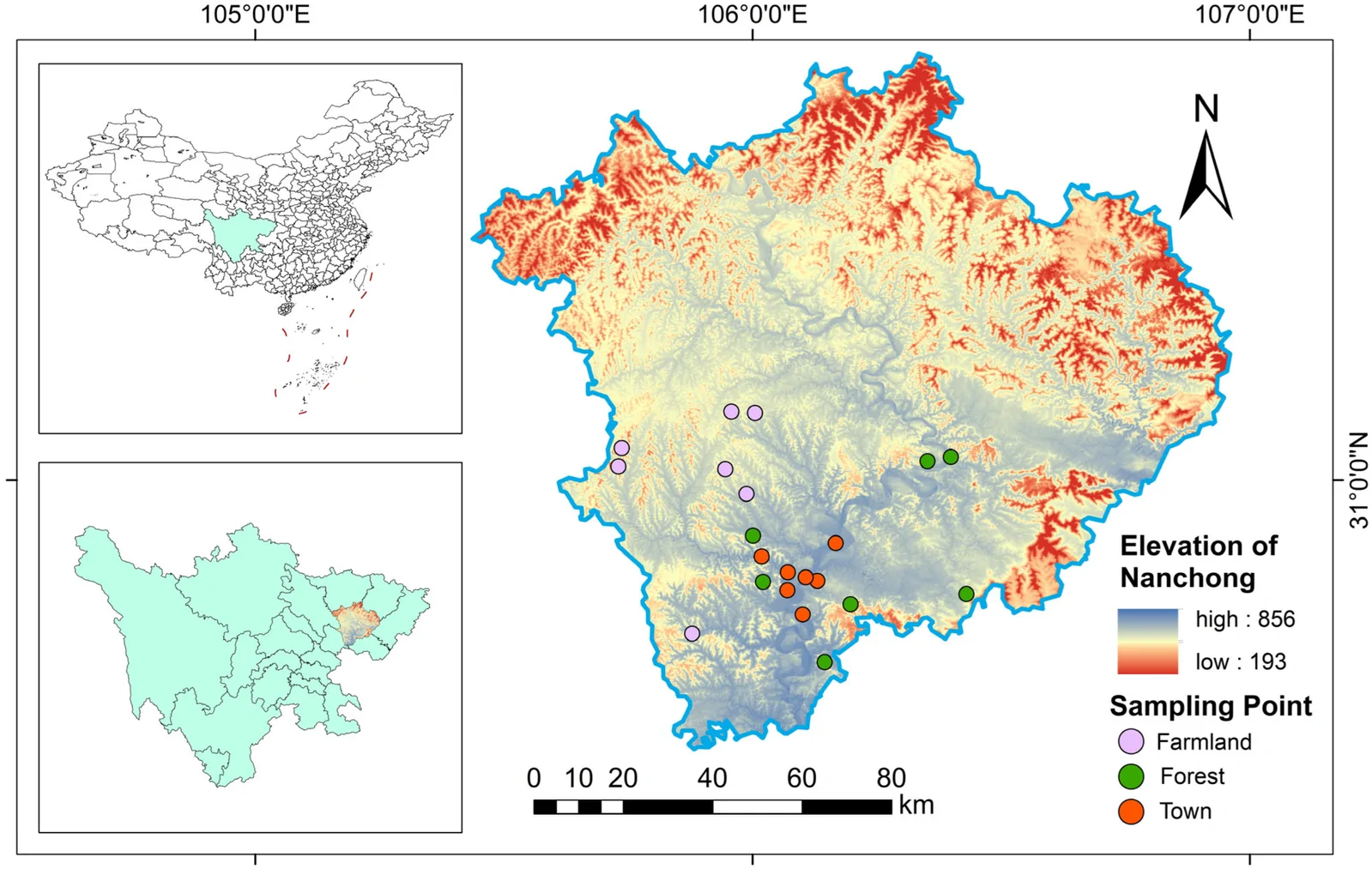
Distribution of sampling points in Nanchong City.

The study area is located within Nanchong city, China (Figure 1) (30°35′–31°51′ N, 105°27′–106°58′ E), along the middle reaches of Jialing River. It has a mid-subtropical humid monsoon climate, characterized by mild winters, early springs, hot summers and autumn rainfall. The landscape is dominated by hills with elevation ranging from 256 m to 889 m. Mean annual temperature is ca. 17.1 °C, with ≥10 °C accumulated temperature of 5500 °C. Annual precipitation averages 1016.8 mm, mostly falling between April and October. Secondary cypress woodlands represent the dominant forest type, while rice and citrus are major crops in intermountain valleys, with a frost-free period of approximately 355 days [45].

**Figure 2:**
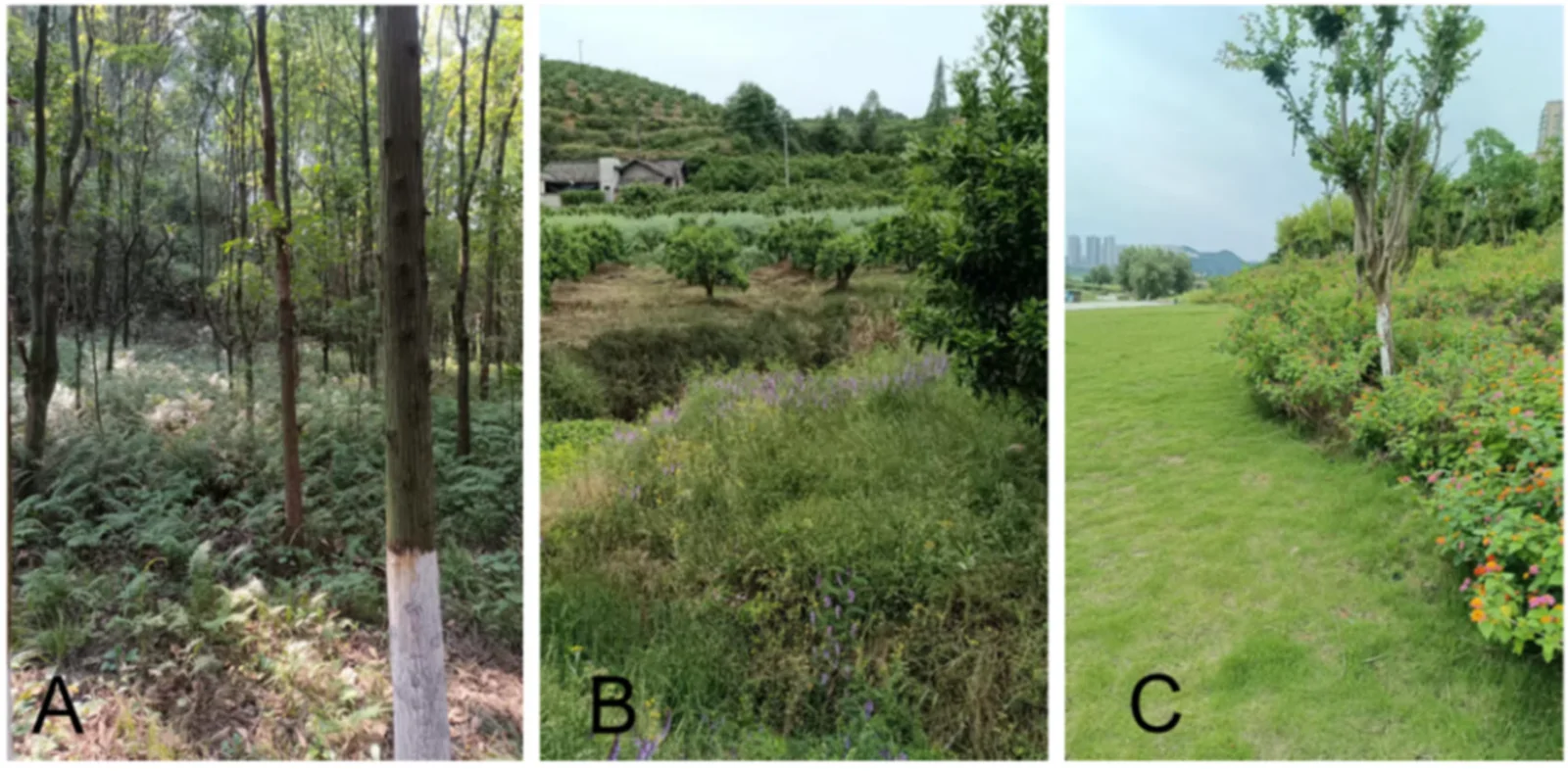
Three types of land use in Nanchong City: (**A**) forest; (**B**) farmland; (**C**) town.

Three representative land-use types (forest, cropland, and urban habitat) were selected as research sites across Nanchong. Seven transects (500 m long × 5 m wide) were established within each habitat type, yielding a total of 21 transects (Figure 1). The latitude and longitude of each transect midpoint were recorded for spatial localization. Urban sampling sites covered flower-rich urban parks, riparian zones, and roadside greenbelts; cropland sites included cultivated farmlands and orchard edges; and forest sites were situated on densely wooded hillsides (Figure 2).

### 2.2. Transect Counts

Pollinator surveys were carried out monthly from April to September in 2024 and 2025 via standardized transect sampling. Surveys were performed only on clear, windless days between 09:00 and 18:00, with investigators walking slowly and uniformly along each transect at a constant speed of 2.5 km/h. Butterflies and bumblebees encountered within a 2.5 m lateral buffer on either side of the transect and a 5 m vertical height range were documented by digital photography and aerial net sweeping [46,47]. The species richness, abundance, and flower-visiting frequency of bees and hoverflies were counted within each 1 × 2 m sampling quadrat, with one sample quadrat for every transect.

We recorded pollinator species, individual abundance, and co-occurring flowering plant species for each transect on a monthly basis. Insect specimens that could not be identified in the field were preserved and transported to the laboratory for taxonomic verification. For available resource and environmental factor data, see Appendix B.

All recorded pollinators were categorized into four functional groups: butterflies, bees, hoverflies, and other pollinators. Butterfly identification followed authoritative taxonomic references including *Monograph of Chinese butterflies* [48], *Butterflies of China* [49] and *Chinese Insects Illustrated* [50]. Bee and hoverfly specimens were identified by professional taxonomic specialists.

### 2.3. Data Analysis

#### 2.3.1. Alpha Diversity of Pollinator Communities

Five widely used α-diversity indices were calculated to characterize pollinator assemblages, with formulas defined as follows:

Shannon–Wiener diversity index: H′ = −∑(N_i_/N)ln(N_i_/N) (1)

Simpson’s diversity index: D = 1 − ∑(N_i_/N)^2^ (2)

Berger–Parker dominance index: D′ = Nmax/N(3)

Pielou evenness index: J = H′/ln S(4)

Margalef richness index: R = (S − 1)/ln N(5)

where N_i_ = number of individuals of the i-th pollinator species, N = total number of pollinator individuals, Nmax= individual abundance of the dominant species, and S = total number of species.

Normality tests were performed prior to inter-habitat comparisons. One-way analysis of variance (ANOVA) was applied for datasets conforming to normal distribution and homogeneity of variance, while the non-parametric Kruskal–Wallis H test was adopted for non-normal data to detect disparities in pollinator richness and α-diversity indices across three land-use types (forest, cropland, urban habitat).

Non-metric multidimensional scaling (NMDS) based on Bray–Curtis dissimilarity matrices was used to visualize compositional dissimilarities of pollinator communities among habitat types. The stress value was used to evaluate the goodness-of-fit of NMDS ordination:

Stress < 0.05: excellent fit with high interpretability;
0.05 < stress < 0.1: good fit with reliable ecological reference;
0.1 < stress < 0.2: marginal fit with limited explanatory power;
0.2 < stress < 0.3: poor fit without valid ecological interpretation.

#### 2.3.2. Environmental Factor Analysis

Environmental factors refer to climatic, topographic and land-cover variables that directly or indirectly regulate insect growth, reproduction, spatial distribution and floral-visiting behavior. Based on GPS coordinates of each transect midpoint, ArcGIS10.8 software was used to extract climatic and topographic variables from the Earth Resource GIS Cloud Platform (www.gis5g.com).

Bivariate regression models were first established to quantify pairwise correlations between each environmental variable and diversity indices. Redundancy Analysis (RDA) was further conducted to disentangle the combined effects of multi-environmental predictors on the spatial patterns of pollinator diversity.

#### 2.3.3. Plant–Pollinator Pollination Network Analysis

**Figure 3:**
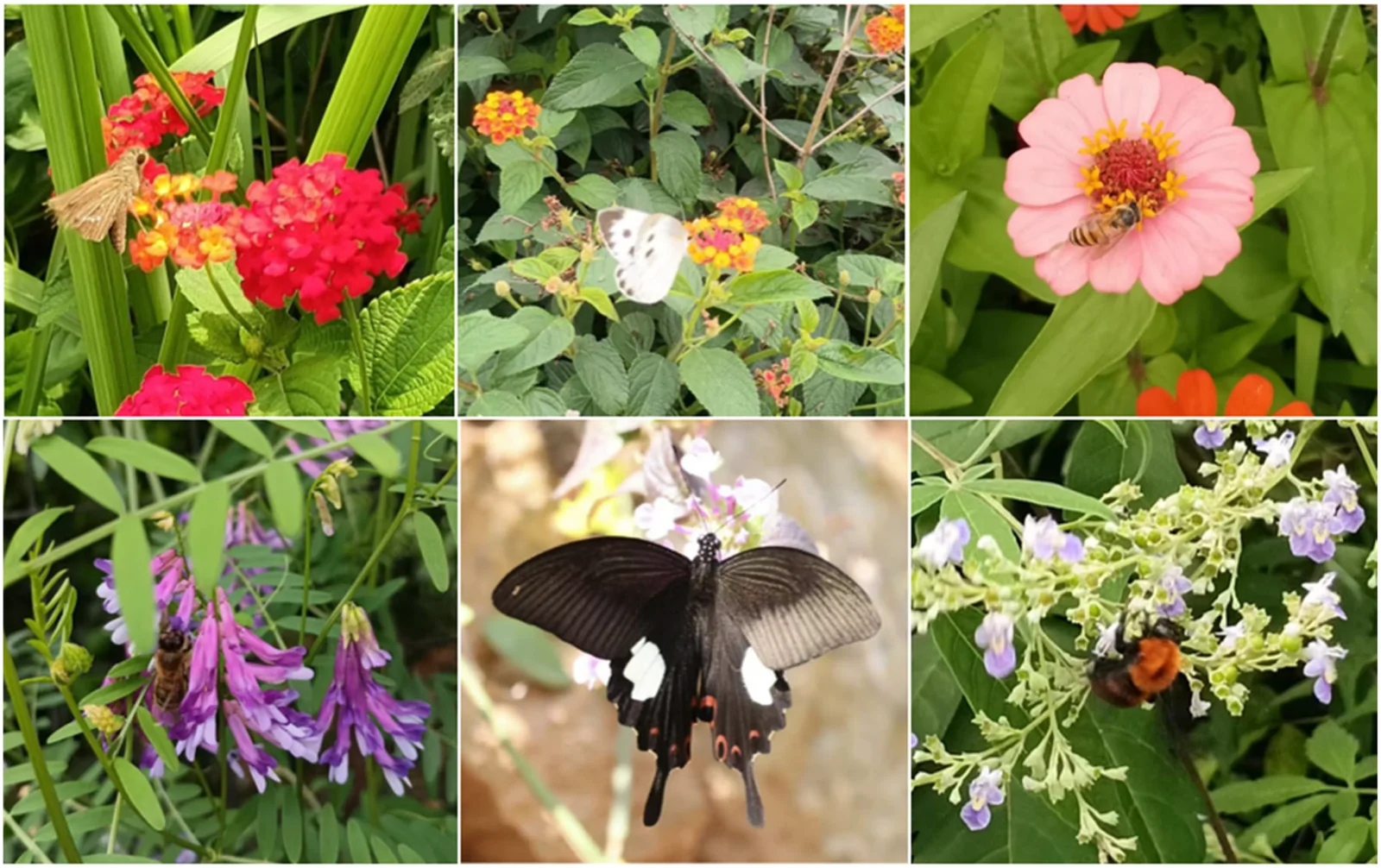
Pollinating insect–nectar plant network.

Nectar-plant density of herbaceous flowers was surveyed using 1 × 2 m sampling quadrats placed in flower-rich zones along each transect, whereas 5 × 5 m sampling quadrats were used for shrub flowers. We recorded the species richness and abundance of flowering plants. Pollinator flower-visiting frequency was observed for 6 min within each 1 × 2 m quadrat. We recorded pollinator taxa and corresponding floral visitation frequencies. A single valid visit was recorded when any body part of an insect contacted pollen or stigmas of focal flowers (Figure 3). Repeated floral-visiting surveys were conducted along each transect following the phenological sequence of flowering plants. Total visitation frequencies of each plant–pollinator pair were accumulated across the entire growing season to construct quantitative weighted bipartite pollination networks.

The network level function embedded in the R4.3.3 bipartite package was used to compute global network-level metrics [51], including connectance, weighted nestedness (wNODF) [52], network-wide specialization (H_2′) [53], interaction dependence asymmetry [54], modularity (Q) [55], network robustness [56], and total number of interaction links [57].

#### 2.3.4. Data Management and Visualization

All raw field data, derived diversity indices and interaction matrices were collated in Microsoft Excel 2019. All statistical analyses and network constructions were implemented in R v4.3.3. Final figures and vector artwork were polished with Adobe Illustrator 2025.

## 3. Results

### 3.1. Pollinator Community Composition of Different Land-Use Types

A total of 3686 pollinator individuals belonging to 99 species, 62 genera and 17 families were collected along 21 transects in Nanchong City, including 2243 butterflies (59 species), 1284 bees (24 species), 119 hoverflies (9 species), and 40 individuals of other taxa (7 species) (Table 1). At the family level, Nymphalidae dominated species richness in forests and farmland. Apidae showed the maximum abundance in forests (382 individuals) and urban areas (313 individuals), whereas Pieridae was most abundant in farmland (637 individuals). Urban pollinator community also contained fewer auxiliary taxa.

### 3.2. Pollinator Community Diversity Across Different Land-Use Types

**Figure 4:**
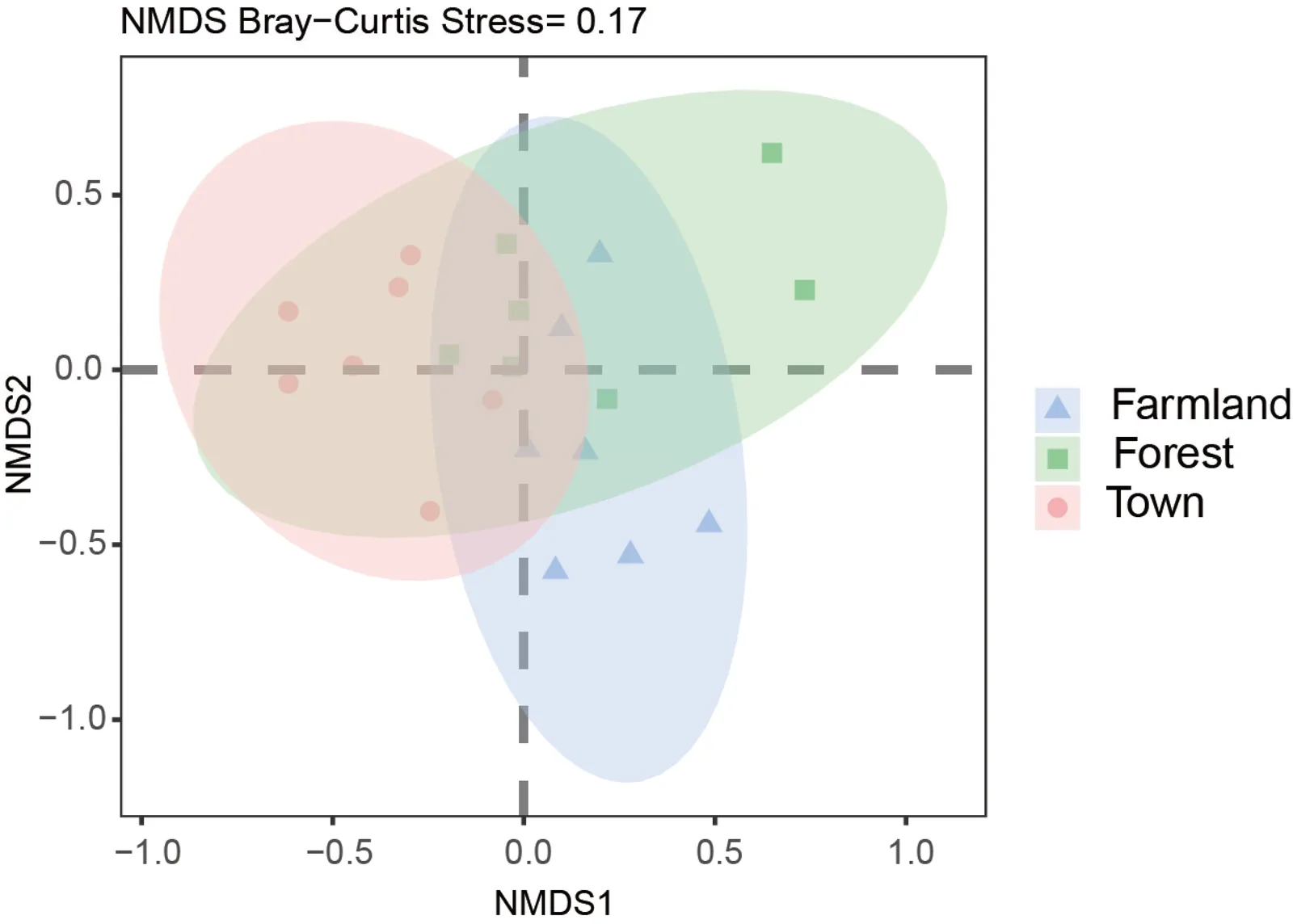
Non-metric multidimensional scaling analysis of pollinator communities in different land-use patterns in Nanchong. Colored ellipses represent the distribution range of samples for each habitat group; The farther the ellipse separates, the greater the difference in community between groups; Elliptical overlap represents high similarity in community composition. The closer the sample distance, the more similar the species composition of the community; The farther the distance, the greater the difference in species composition.

The NMDS stress value was 0.17 (<0.2), indicating credible ordination results (Figure 4). Pollinator communities of forest, farmland and urban habitats were distinctly separated with partial overlapping, suggesting significant structural differences among the three assemblages and species spillover from surrounding habitats. Forest habitats covered the majority of pollinator species, acting as the core pollinator resource bank.

**Figure 5:**
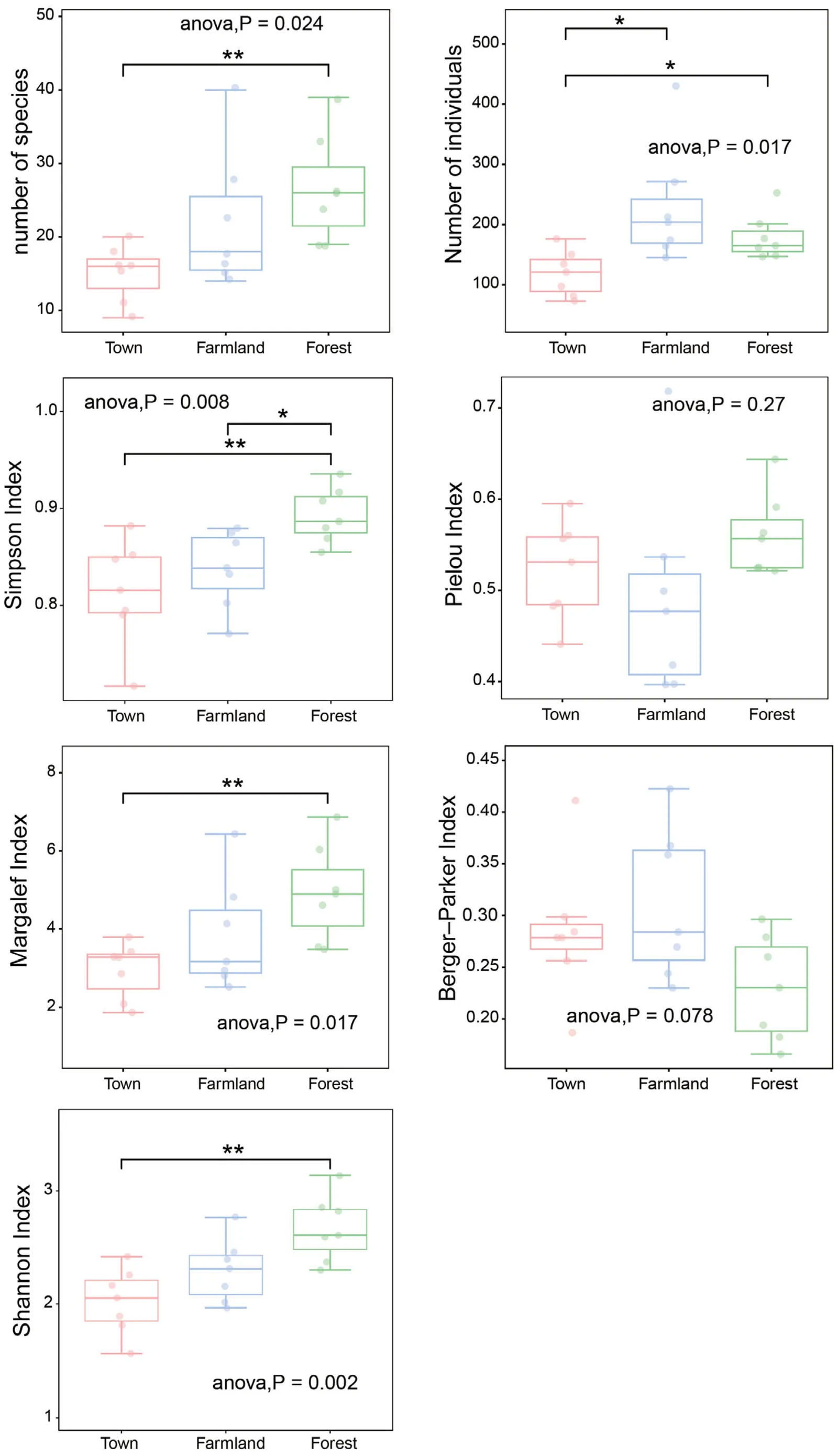
Differences in pollinator insect communities under different land-use types. * Indicates a significant difference at the 0.05 level, and ** indicates an extremely significant difference at the 0.01 level.

One-way ANOVA showed that species richness, individual abundance, Margalef richness, and Simpson and Shannon–Wiener diversity indices differed extremely significantly among the three land-use types (*p* < 0.01), while Pielou evenness and Berger–Parker dominance indices had no significant differences (Figure 5). Pairwise comparison results were as follows: Extremely significant differences in species richness, Simpson index, Shannon index and Margalef index were only detected between urban and forest habitats (*p* < 0.01). Urban areas had significantly lower individual abundance than farmland and forests (*p* < 0.05). Farmland showed significantly lower Simpson diversity than forests (*p* < 0.05), with no significant differences in other pairwise comparisons.

As illustrated in Figure 5, pollinator community metrics differed substantially across the three land-use types. One-way analysis of variance (ANOVA) revealed highly significant inter-habitat differences (*p* < 0.01) in total species richness, individual abundance, Margalef richness index, Simpson diversity index and Shannon–Wiener diversity index. In contrast, no significant variations were detected for the Pielou evenness index and Berger–Parker dominance index among habitats.

Post hoc multiple comparisons for each index are summarized below:

(1) Total species richness: Highly significant divergence was found between urban and forest habitats (*p* = 0.005 < 0.01), while pairwise comparisons of urban vs. cropland and forest vs. cropland showed non-significant differences (*p* = 0.20 and *p* = 0.20, respectively).
(2) Individual abundance: Significant differences existed between urban and cropland (*p* = 0.011 < 0.05), as well as urban and forest habitats (*p* = 0.021 < 0.05). Abundance did not differ significantly between forests and croplands (*p* = 0.31).
(3) Simpson diversity index: Urban and forest sites differed highly significantly (=0.007 < 0.01), and croplands and forests showed a significant disparity (*p* = 0.015 < 0.05); no statistical difference was observed between urban and cropland habitats (*p* = 0.52).
(4) Shannon–Wiener diversity index: Only the urban–forest comparison yielded a highly significant difference (*p* = 0.005 < 0.01), with non-significant contrasts between urban–cropland and forest–cropland pairs.
(5) Margalef richness index: A highly significant gap occurred between urban and forest habitats (*p* = 0.005 < 0.01), whereas urban vs. cropland (*p* = 0.44) and forest vs. cropland (*p* = 0.097) comparisons were non-significant.

### 3.3. Effects of Environmental Variables on Pollinator Community Diversity

#### 3.3.1. Bivariate Regression Analysis

**Figure 6:**
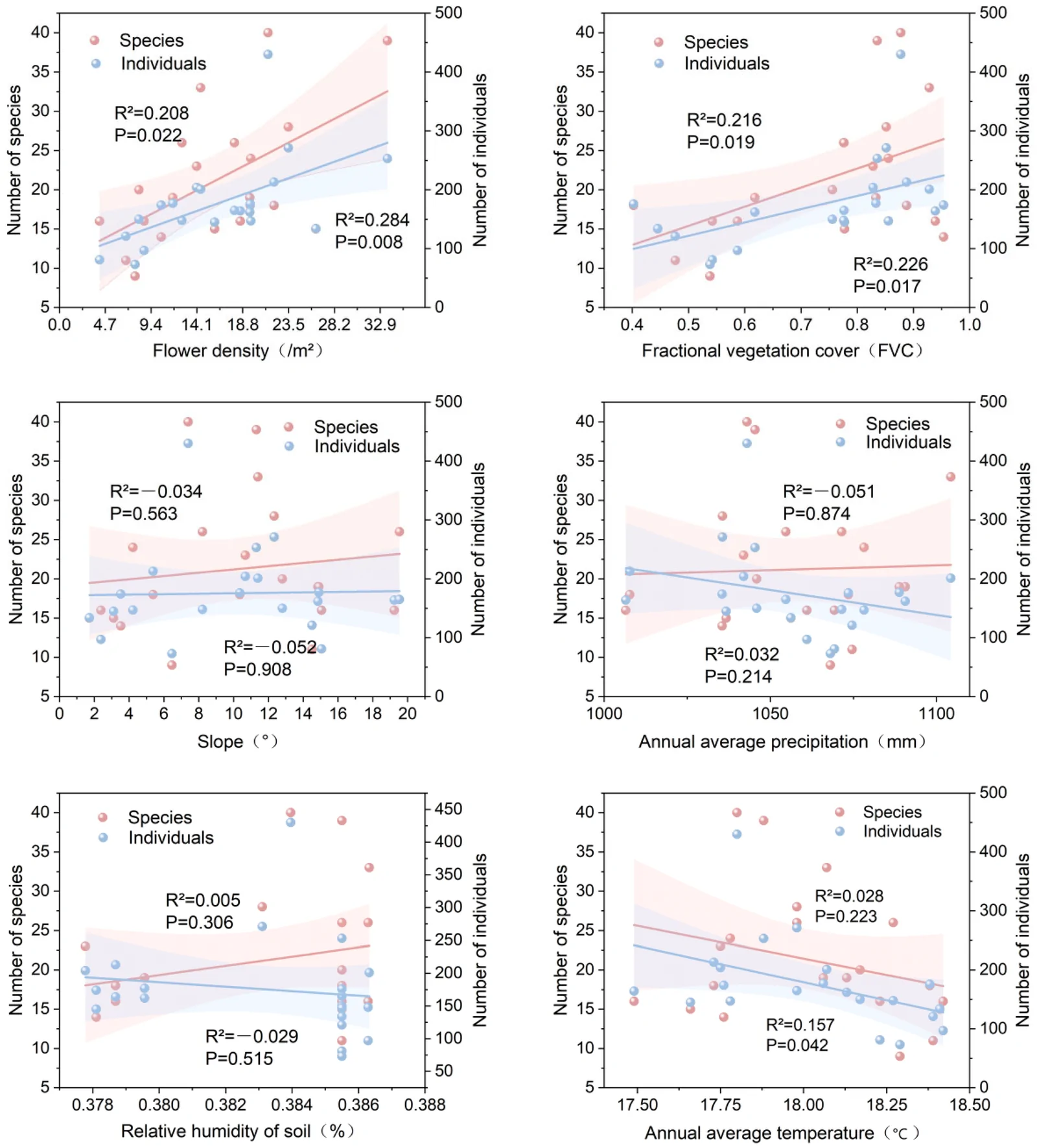

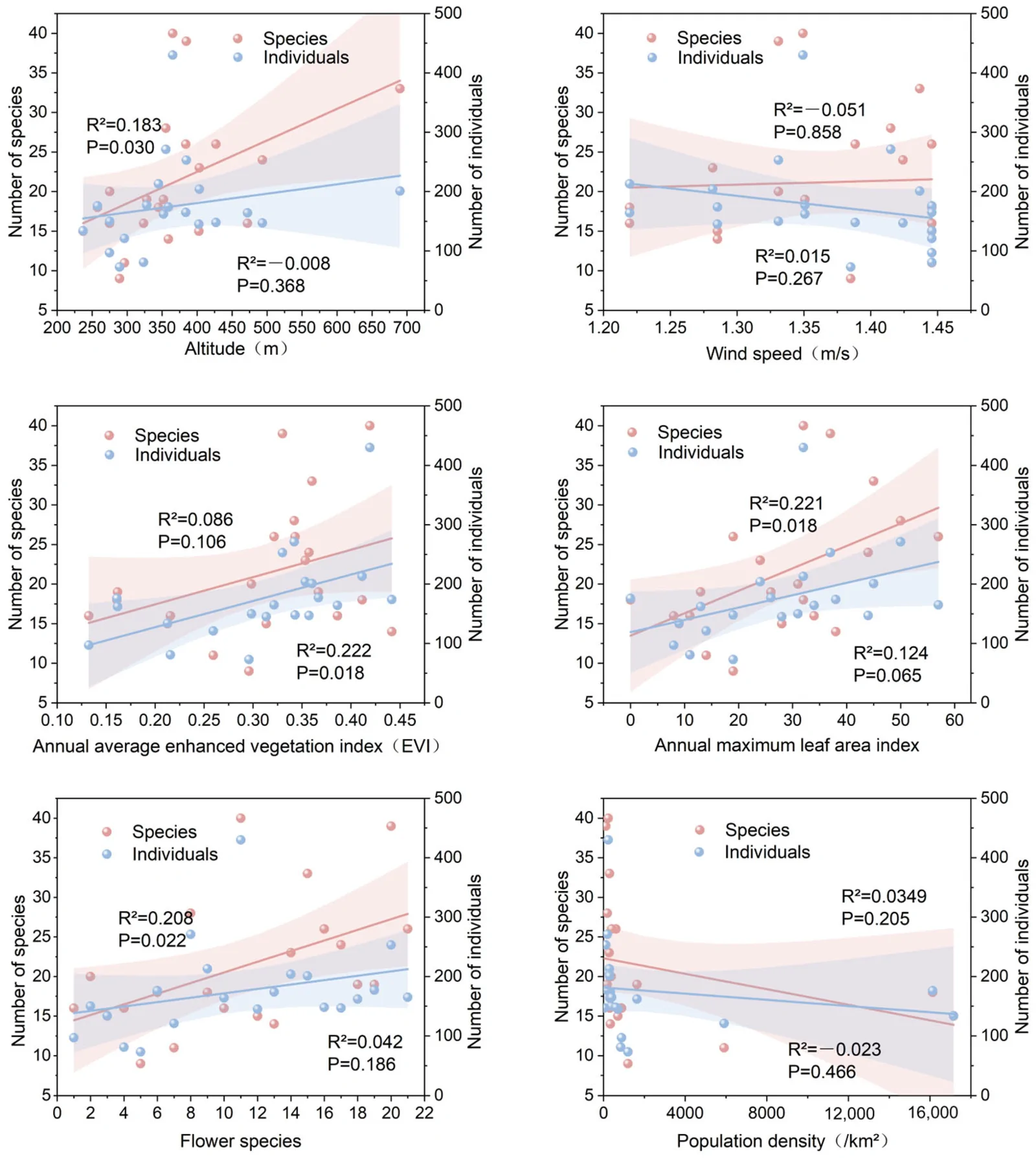
Linear fitting of species and individual numbers of pollinators with environmental factors.

Bivariate regression models were applied to quantify the relationships between environmental predictors and pollinator species richness as well as individual abundance (Figure 6). The strength and significance of correlations varied markedly across environmental variables:

(1) Flower richness was significantly and positively correlated with pollinator species richness (R^2^ = 0.208, *p* = 0.022 < 0.05).
(2) Flower density showed a significant positive correlation with species richness (R^2^ = 0.208, *p* = 0.022 < 0.05), and a highly significant positive correlation with individual abundance (R^2^ = 0.284, *p* = 0.008 < 0.01).
(3) Elevation was positively correlated only with species richness (R^2^ = 0.183, *p* = 0.030 < 0.05), with no significant association with abundance.
(4) Maximum leaf area index (LAImax) exhibited a significant positive correlation solely with species richness (R^2^ = 0.221, *p* = 0.018 < 0.05), showing no detectable effect on abundance.
(5) Fractional vegetation cover (FVC) was significantly positively associated with both species richness (R^2^ = 0.216, *p* = 0.019 < 0.05) and individual abundance (R^2^ = 0.226, *p* = 0.017 < 0.05).
(6) Annual mean enhanced vegetation index (EVI) correlated positively only with individual abundance (R^2^ = 0.222, *p* = 0.018 < 0.05), with no significant link to species richness.
(7) Annual mean temperature had a significant negative correlation with individual abundance (R^2^ = 0.157, *p* = 0.042 < 0.05), and exerted no statistically significant influence on species richness.

Five predictors, including wind speed, human population density, relative soil humidity, slope gradient and annual precipitation, displayed no significant correlations with either pollinator species richness or total abundance.

#### 3.3.2. Redundancy Analysis (RDA) Ordination

**Figure 7:**
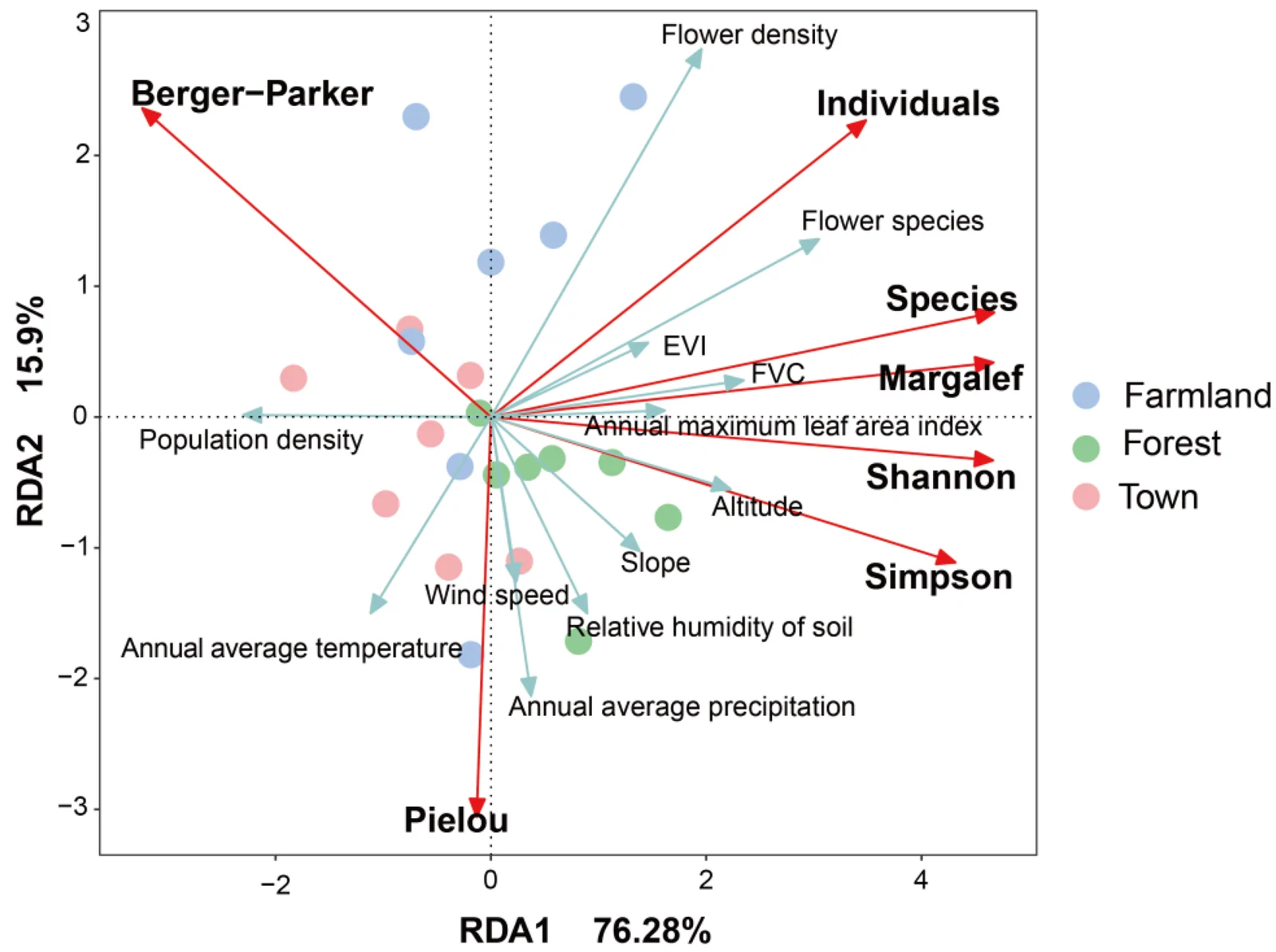
RDA of pollinator insect diversity and environmental factors. The red arrow represents diversity index and quantity, while the light green arrow represents environmental factors. The longer the arrow, the greater the impact of the factor, while the shorter the arrow, the smaller the impact. When the angle between two variables forms an acute angle, the two factors are positively correlated; When two variables form an obtuse angle, the two factors are negatively correlated. When RDA > 0, it is positively correlated with factors; When RDA < 0, it is negatively correlated with factors. If the sample point is closer to the arrow, it indicates that the environmental and diversity characteristics of the sample are more similar to this factor.

Redundancy Analysis (RDA) was further used to disentangle the combined effects of multi-environmental variables on pollinator diversity patterns (Figure 7).

For the first RDA axis, ten variables (flower density, flower richness, annual EVI, FVC, LAImax, elevation, slope, wind speed, relative soil humidity, annual precipitation) showed positive correlations, while human population density and annual mean temperature were negatively correlated. Human population density had the highest correlation coefficient with Axis 1, indicating that Axis 1 primarily represented the gradient of anthropogenic disturbance; annual precipitation had the weakest correlation with this axis.

For the second RDA axis, six variables (flower richness, flower density, annual EVI, FVC, LAImax, human population density) were positively correlated, whereas elevation, slope, wind speed, relative soil humidity, annual precipitation and annual mean temperature were negatively correlated. Annual precipitation exhibited the strongest correlation with Axis 2, which thus represented the regional moisture gradient; human population density had the lowest correlation with Axis 2.

Correlations between each community metric and environmental variables were summarized as follows:

(1) Individual abundance: positively correlated with seven variables (e.g., flower richness, flower density, FVC, elevation) and negatively correlated with five variables (e.g., wind speed, soil humidity);
(2) Species richness: positively correlated with eight variables (e.g., annual EVI) and negatively correlated with four variables (e.g., annual temperature);
(3) Margalef richness index: positively associated with ten environmental variables and negatively associated with two variables;
(4) Shannon and Simpson diversity indices: both positively correlated with ten variables and negatively correlated with the remaining two variables;
(5) Pielou evenness index: negatively correlated with four floral/vegetation variables (flower density, flower richness, annual EVI, FVC) and positively correlated with all other predictors;
(6) Berger–Parker dominance index: only positively correlated with human population density and flower density, and negatively correlated with all other environmental factors.

The vector length of each environmental variable in the RDA biplot reflected its explanatory power for pollinator community variation. Flower richness and flower density had the longest vectors, demonstrating that they were the dominant drivers shaping pollinator assemblages. By contrast, annual EVI and LAImax possessed the shortest vectors and exerted the weakest influence on community structure.

### 3.4. Seasonal Dynamics of Plant–Pollinator Mutualistic Networks

**Figure 8:**
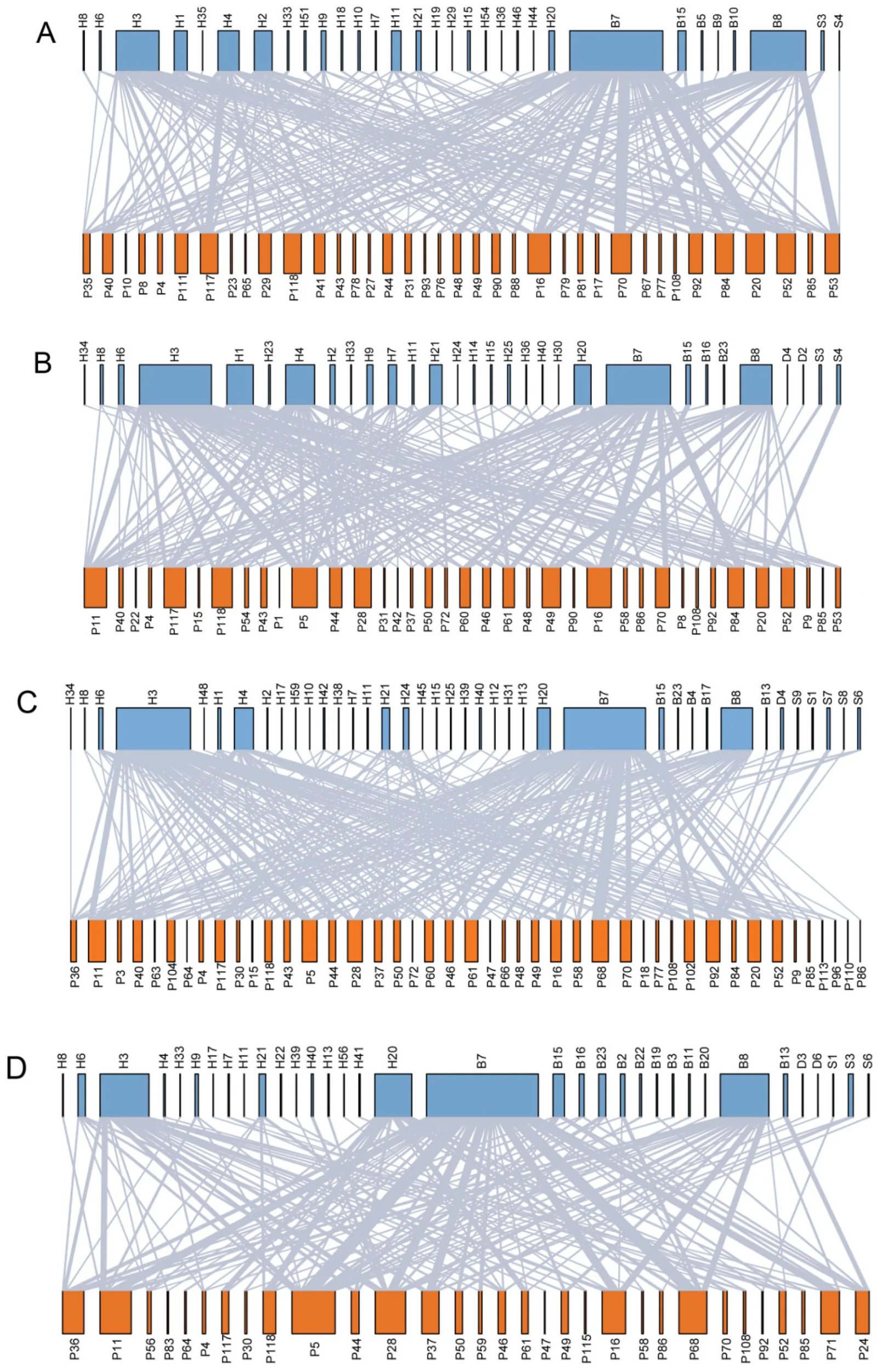

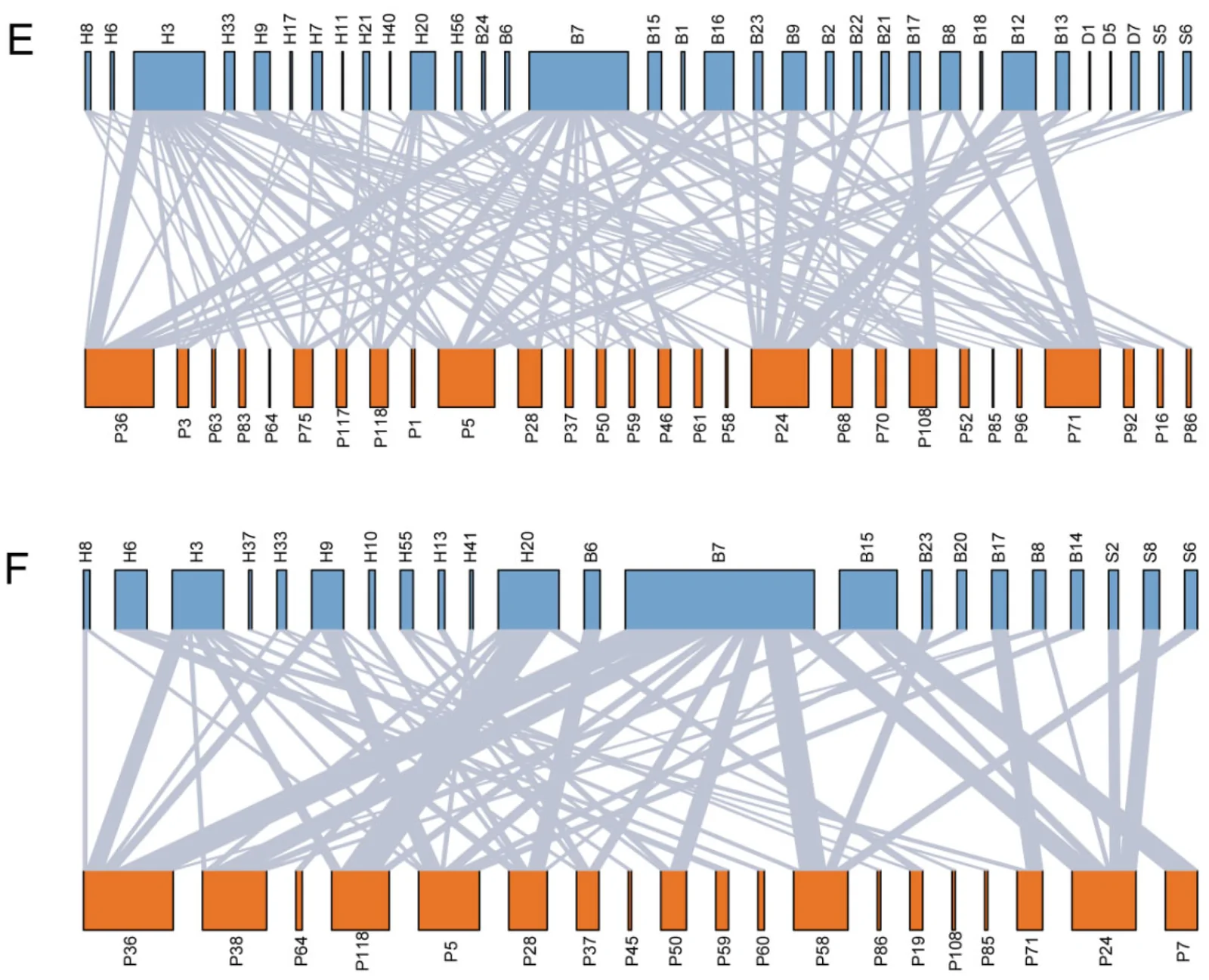
Bipartite plant–pollinator network by month in Nanchong. (**A**–**F**) respectively represent the months of April to September. Note: The blue part represents pollinating insects, the orange part represents plants, and the gray line in the middle represents their interaction.

**Figure 9:**
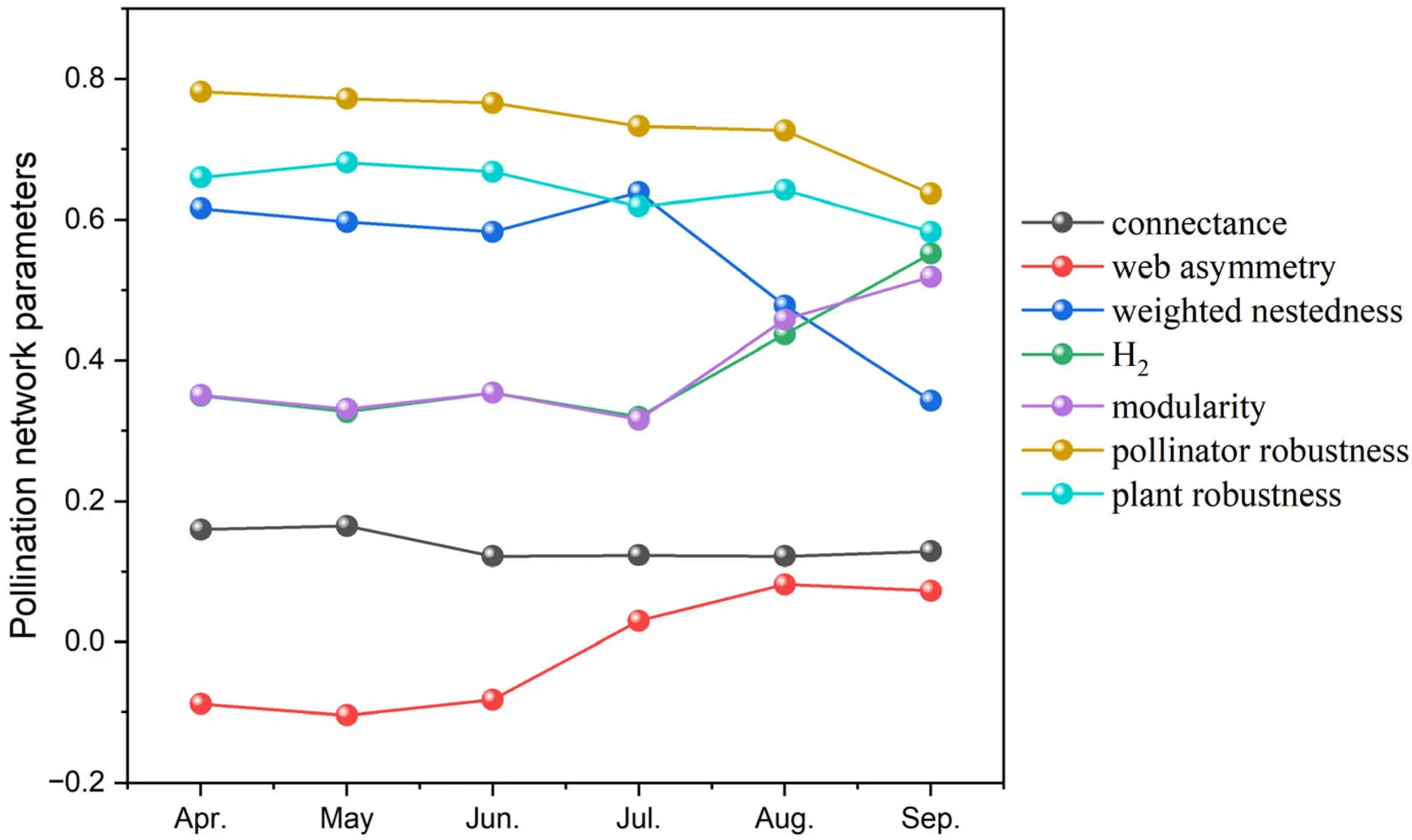

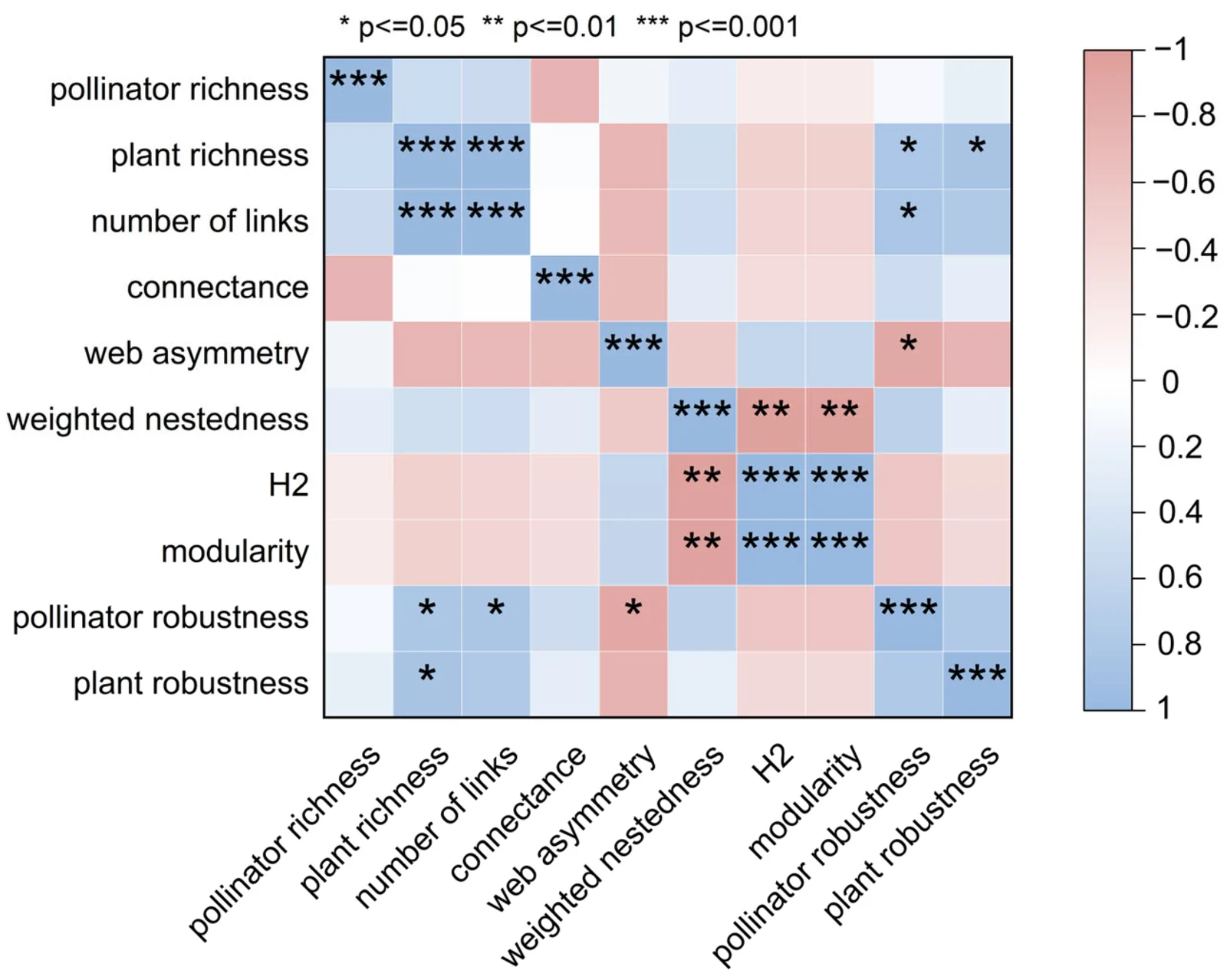
Dynamic changes in pollination network parameters (**up**) and visualization heatmap of pollination network parameters (**down**).

Monthly bipartite plant–pollinator interaction networks were constructed from April to September (Figure 8). Global network-level metrics were calculated for each month to reveal seasonal shifts in pollination network architecture across the growing season (Figure 9). Seasonal trends of each structural index were summarized as follows:

(1) Connectance: Connectance remained stable from April to May, peaked in June, and then declined continuously to the annual minimum in September. Overall connectance was consistently low throughout the season, with a slight rebound observed in September.
(2) Interaction asymmetry: Negative values occurred between April and June, indicating higher plant species richness relative to pollinators, with the most negative value recorded in May. The index turned positive and increased steadily from July to September, revealing that pollinator richness exceeded plant richness in late summer and autumn. This pattern demonstrated that flowering plant communities declined faster than pollinator assemblages along the phenological gradient.
(3) Weighted nestedness (wNODF): Weighted nestedness showed mild seasonal fluctuation and reached its maximum value in July, maintaining relatively stable levels across all months.
(4) Network-wide specialization (H_2′) and modularity (Q): Both indices increased progressively over time and peaked simultaneously in September. This upward trend indicated rising specialization of pairwise plant–pollinator interactions as flowering progressed; interactions were partitioned into distinct, tightly connected but isolated modules in late season.
(5) Network robustness: Pollinator-layer robustness was consistently higher than plant-layer robustness across all months, demonstrating that pollinator assemblages possessed stronger resistance to the loss of flowering plant resources, whereas plant communities were more vulnerable to pollinator decline. Robustness of both trophic layers declined month by month, and the entire network exhibited the weakest stability and highest fragility in September.

Correlation heatmaps were generated to quantify pairwise associations among all network structural parameters:

(1) Plant species richness was significantly positively correlated with plant-layer robustness (R = 0.812) and pollinator-layer robustness (R = 0.841), and exhibited an extremely strong positive correlation with total interaction links (R = 0.986);
(2) Connectance was significantly positively associated with pollinator-layer robustness (R = 0.829);
(3) Interaction asymmetry showed a significant negative correlation with pollinator-layer robustness (R = −0.886);
(4) Weighted nestedness had extremely significant negative correlations with both network-wide specialization and modularity (R = −0.943), (R = −0.943);
(5) Network-wide specialization was perfectly positively correlated with modularity (R = 1).
(6) Total floral visitation frequency of pollinators had an extremely significant linear relationship with flowering plant species richness (R^2^ = 0.793), (*p* < 0.001), confirming that floral diversity acts as the primary driver regulating overall pollinator foraging activity.

## 4. Discussion

This study focused on the effects of land-use patterns on the structure and diversity of pollinator communities in Nanchong City. We clarified the regulatory mechanisms of vegetation, topography, and other environmental factors on pollinator assemblages, revealed the seasonal dynamics of plant–pollinator interaction networks, and proposed targeted pollinator conservation and utilization strategies based on regional agricultural characteristics. The findings provide a theoretical basis for regional pollinator biodiversity conservation and the maintenance of agricultural pollination ecosystem services.

### 4.1. Effects of Land-Use Patterns on Pollinator Communities

Habitat loss and degradation are primary threats to global biodiversity, substantially altering species distribution, population abundance, spatial patterns, and functional composition of local communities [58,59,60]. Consistent with previous studies, our results showed significant differences in pollinator community characteristics among different land-use types, with obvious divergence in species richness, population abundance, community dominance, and Shannon–Wiener diversity index. This confirms that land-use pattern is a key anthropogenic driver shaping community differentiation and regulating the species composition and abundance of pollinators. The availability of critical resources is one of the major drivers of butterfly community composition and distribution patterns [61]. In particular, nectar resources are key to ‘fuel’ flight activity for adults which is highly relevant for mating and egg deposition, while larval host plants are key for larval development and viability [61,62].

Forest ecosystems are the most important habitats of biodiversity. Approximately 80% of terrestrial biodiversity relies on forest ecosystems for survival [63]. Forests exhibited the highest pollinator species richness and Shannon–Wiener diversity index with the lowest community dominance, contributing to the most balanced pollinator community structure due to complex vegetation architecture, sufficient food resources, and stable habitat conditions. Croplands showed intermediate levels of species richness, population abundance, and diversity but the highest community dominance. Urban habitats supported the lowest pollinator species diversity and abundance among the three habitat types. Such distribution patterns are mainly determined by habitat filtering effects; differences in resource availability, spatial configuration of habitat patches, and anthropogenic disturbance intensity jointly drive pollinator community divergence across habitats [4].

Forest habitats possess high vegetation coverage and abundant host and nectar plant resources [64], as well as complex vertical vegetation layers (arbor, shrub, and herb layers) and highly connected habitat patches. Diverse plant resources provide continuous food supplies for pollinator larvae and adults. Multi-layered vegetation structures offer differentiated microhabitats for pollinators, especially butterflies, to roost, reproduce, and forage [34,35]. High patch connectivity reduces dispersal resistance, facilitates population exchange, and effectively sustains the stability and diversity of pollinator communities [65].

Croplands maintained moderate pollinator diversity and shared numerous species with forests, acting as critical ecological corridors for cross-habitat pollinator dispersal. Most farmlands in Nanchong are adjacent to mountain forests, promoting species overlap between cropland and forest pollinator assemblages. In addition, the large-scale cultivation of cruciferous crops and the widespread artificial apiculture significantly promote the population proliferation of *Pieris rapae* and *Apis cerana*, resulting in the highest overall pollinator abundance in croplands. However, intensive agricultural management, especially the extensive application of pesticides in orchards and vegetable fields, kills not only pests but also wild pollinators, restricting the improvement of cropland pollinator diversity [1].

Urban habitats harbored significantly fewer pollinator species and lower abundance than forests and croplands, mainly due to artificial and homogenized habitat structure and resource allocation. Urban ornamental plants are artificially unified with limited species and highly concentrated flowering periods, which only sustain a small number of adapted pollinators temporarily [1]. Pollinator populations decline rapidly after flowering seasons, leading to reduced overall community diversity [66]. Furthermore, urban habitats are highly fragmented, isolated, and poorly connected, limiting pollinator dispersal among green patches and inhibiting the maintenance and renewal of urban pollinator communities.

### 4.2. Dominant Pollinator Groups in Different Habitats

Lepidoptera dominated the pollinator communities in Nanchong City, followed by Hymenoptera and Syrphidae. This community composition is closely related to regional agricultural intensification and the large-scale development of artificial beekeeping [67].

At the family level, Nymphalidae exhibited the highest species richness in forest habitats, which is consistent with previous regional pollinator studies. Most Nymphalidae species are generalists with wide host plant ranges and strong dispersal abilities, enabling them to efficiently acquire food resources and adapt to diverse habitat patches in complex forest ecosystems [68].

Apidae and Pieridae were the dominant groups in croplands. Large-area cruciferous crop cultivation and artificial beekeeping provide sufficient nectar and pollen resources for *Apis cerana* and *Pieris rapae*, promoting their population proliferation and dominating the farmland pollinator assemblages, which substantially increases overall pollinator abundance in agricultural habitats.

Restricted by single plant species, seasonal flowering phenology, limited habitat area, and high patch isolation, urban habitats present the lowest pollinator diversity with loose community structure and no stable dominant pollinator groups, resulting in poor community stability and disturbance resistance [68].

### 4.3. Key Factors Affecting Pollinator Diversity

The decline of pollinator diversity is driven by the synergistic effects of vegetation characteristics and environmental factors. It is not only directly caused by habitat loss and fragmentation but also indirectly induced by the reduction in nectar plants and wild flower resources [69].

This study verified that landscape-scale vegetation coverage is the core driving factor of pollinator species richness, while wild flower richness and density serve as the most influential variables. Flower richness and density were significantly positively correlated with pollinator species richness, abundance, Shannon diversity index, and Margalef richness index, but negatively correlated with community dominance index. These results indicate that floral resources play a more critical role in regulating local pollinator community assembly compared with other environmental variables, and sufficient wild flower resources can effectively enhance pollinator diversity [70].

Topographic factors also significantly shaped pollinator community structure [4]. Slope was negatively correlated with community dominance, while elevation showed significant positive correlations with the Shannon index and Margalef richness index. High-altitude areas feature complex terrain, high habitat heterogeneity, and low anthropogenic disturbance, supporting richer pollinator species. In contrast, low-altitude regions suffer from intensive land-use transformation and severe habitat homogenization, which reduce local species diversity and further exacerbate the dynamic variation in pollinator species distribution at high elevations [71].

Notably, human population density showed no significant influence on pollinator communities in this study. This may be because population quantity cannot directly reflect the intensity and mode of anthropogenic land disturbance, thereby exerting no direct regulatory effect on pollinator assemblages.

### 4.4. Seasonal Dynamics of Plant–Pollinator Networks

The plant–pollinator interaction networks in Nanchong City exhibited significant seasonal variation in stability, specialization, and modularity. The network presented a consistent seasonal transition pattern: generalized and stable in spring, structurally transitional in early summer, and specialized and fragile in late summer and early autumn.

In spring (April–May), abundant flowering plants provided sufficient food resources, with more plant species than pollinator species. Plant–pollinator interactions were frequent and diverse, forming a distinct nested and generalized network structure. This network possessed high fault tolerance and stability, in which the absence of individual species could be compensated by alternative species, ensuring efficient and stable pollination services during spring.

In early summer (June–July), the number of species and network links reached the annual peak with the largest network scale. During this period, network connectivity gradually decreased while modularity increased, and the interaction pattern shifted from generalized to specialized associations with enhanced interspecific specificity.

In late summer and early autumn (August–September), flowering crops and wild plants gradually withered, causing a drastic reduction in floral resources. Plant species declined faster than pollinator species, resulting in a higher richness of pollinators relative to flowering plants. Resource scarcity drove the formation of highly specialized plant–pollinator mutualisms, generating tightly connected but ecologically isolated functional modules and consequently increasing network specialization and modularity. The pollination network exhibited limited species compensatory capacity during this period. The loss of core plant or pollinator species could disrupt key pairwise interactions and trigger the collapse of local subnetworks, thereby severely degrading regional pollination ecosystem services. In late autumn and winter, floral resources become further depleted, and most wild pollinators enter overwintering dormancy. Nevertheless, artificially reared honeybees (*Apis cerana* and *Apis mellifera*) remain active and forage for nectar on warm and sunny days. Cold-tolerant flowering plants (e.g., *Asteraceae*) provide persistent floral resources throughout the cold season, sustaining overwintering honeybee colonies and maintaining seasonal pollination functions at the regional scale.

Overall, the plant–pollinator interaction network showed strong seasonal dynamics, shifting from a robust generalized structure in spring to a vulnerable specialized structure in late summer and early autumn, with continuous declines in network stability throughout the growing season.

### 4.5. Implications for Pollinator Conservation and Agricultural Production

Nanchong City has a large planting area and high industrial value of pollinator-dependent crops, with oilseed rape, citrus, and other fruits and vegetables highly reliant on insect pollination. Pollination services contribute 8–13% of the regional agricultural output value [3]. Therefore, targeted pollinator conservation and management during agricultural production are crucial for stabilizing crop yield, improving fruit quality, and promoting sustainable green agriculture.

Vegetation coverage and wild floral resources are core determinants of pollinator diversity. Protecting natural vegetation and restoring wild pollinator habitats are fundamental measures to sustain stable pollinator communities. Reserving idle land around orchards and vegetable fields to maintain wild flower communities can provide continuous food resources and habitats for pollinators, effectively improving pollinator diversity in agroecosystems and balancing agricultural production and biodiversity conservation.

Considering the seasonal dynamics of pollination networks, the stable generalized network in spring guarantees high pollination efficiency, whereas the specialized and fragile network in late summer and autumn represents a critical conservation window. Therefore, conservation management should prioritize the retention of core wild flowering species with high network connectivity, especially autumn and winter flowering plants, to fill the seasonal food gap after crop flowering. Sustained nutritional supply for overwintering pollinators helps maintain regional pollinator populations and community stability and realizes year-round stable pollination ecosystem services.

## 5. Conclusions

Habitat type exerts a significant influence on pollinator community structure, with forests supporting substantially higher pollinator diversity than croplands and urban areas. Vegetation coverage and nectar plant richness are the key environmental factors shaping pollinator diversity. Plant–pollinator interaction networks exhibit distinct seasonal dynamics, shifting progressively from generalized to specialized structures throughout the year. Reducing pesticide application and developing pollinator-friendly agricultural systems are critical strategies for the conservation and sustainable management of pollinator communities. Furthermore, retaining wild flowering herbs adjacent to pollinator-dependent crops and preserving autumn- and winter-blooming plant species can maintain continuous floral resources. This practice effectively sustains pollinator populations, promotes pollinator richness, stabilizes community assembly, and enhances regional pollination ecosystem services.

## Figures and Tables

**Table 1:** Pollinators diversity in three habitats in Nanchong City.

Group	Abundance	Species	Proportion of Abundance%
butterfly	2243	59	60.85
bee	1284	24	34.83
hoverfly	119	9	3.23
other	40	7	1.09
total	3686	99	
farmland	1601	58	43.43
forest	1253	66	33.99
urban	832	43	22.57

## Data Availability

All data are available by request.

## Appendix A. Data of Transect Countsin Three Habitat Type in Nanchong City

**Sample Code**	**Land-Use Type**	**Sample Name**	**Number of** **Species**	**Number of** **Individual**
1	Town	South of the river	16	97
2	Town	Campsite	20	150
3	Town	Qinglan Road	15	134
4	Town	Wanghe 3	16	81
5	Town	Xiaoqiaozi Street	9	73
6	Town	By the school river	18	176
7	Town	Yudai South Road	11	121
8	Farmland	Beside Liechang	28	271
9	Farmland	Rentian Field	18	213
10	Farmland	Renshan Peak	16	164
11	Farmland	Shi kugou	40	430
12	Farmland	Under the peach blossoms	15	145
13	Farmland	Tianma Agriculture	14	174
14	Farmland	Yuandou Garden	23	204
15	Forest	Jincheng Mountain	33	201
16	Forest	Lingshan Half	26	148
17	Forest	Lingshan Peak	24	147
18	Forest	Longjiao Mountain	19	162
19	Forest	On Mount Peng	19	177
20	Forest	Qi pingzhai	39	253
21	Forest	Qi leya	26	165

## Appendix B. Available Resource and Environmental Factor Data

**Sample Code**	**Flower Density**	**Flower Species**	**Fractional Vegetation Cover** **(** **FVC** **)**	**Population Density**	**Slope** **(** **°** **)**	**Annual Average Precipitation** **(** **mm** **)**
1	8.69	11	0.58711	887.223	2.38594	1061
2	8.15	13	0.75575	374.756	12.8101	1045.9
3	26.3	13	0.44541	17160.1	1.72101	1056.2
4	4.12	7	0.54236	844.728	15.0562	1069.3
5	7.76	9	0.53818	1193.19	6.46663	1068.1
6	19.56	16	0.4025	16133.1	10.3678	1073.5
7	6.82	9	0.47641	5910.48	14.5136	1074.6
8	23.48	11	0.85168	186.949	12.3385	1035.6
9	22.01	18	0.88775	287.721	5.38598	1007.7
10	18.55	15	0.93877	303	19.2317	1006.5
11	21.4	23	0.87735	230.356	7.39396	1043
12	15.93	9	0.77748	701.209	3.10963	1036.7
13	10.44	10	0.95361	332.589	3.52041	1035.5
14	14.08	22	0.8283	278.01	10.6784	1042
15	14.48	23	0.92823	289.54	11.3957	1104.3
16	12.57	19	0.7759	608.771	8.21996	1071.5
17	19.64	19	0.8556	51.3314	4.22269	1078.2
18	19.54	20	0.61797	1641.8	14.8511	1090.6
19	11.63	16	0.83341	177.927	14.907	1088.8
20	33.65	26	0.83572	109.289	11.3147	1045.4
21	17.96	18	0.77671	403.03	19.5336	1054.7

### Available Resource and Environmental Factor Data (Continued)

**Sample Code**	**Altitude** **(** **m** **)**	**Annual Average Temperature**	**Wind Speed (m/s)**	**Annual Average Enhanced Vegetation Index**	**Annual Maximum Leaf Area Index**	**Relative Humidity of Soil**
1	274.9	18.42	1.44575	0.13238	8	0.38629
2	275.1	18.17	1.331	0.29843	31	0.3855
3	237	18.41	1.44575	0.21275	9	0.3855
4	323.5	18.23	1.44575	0.2157	11	0.3855
5	289.2	18.29	1.385	0.29595	19	0.3855
6	257.6	18.38	1.44575	0.16106	0	0.3855
7	296.3	18.39	1.44575	0.25938	14	0.3855
8	355.4	17.98	1.415	0.34205	50	0.3831
9	344.9	17.73	1.2195	0.41132	32	0.37869
10	472	17.49	1.2195	0.3862	34	0.37869
11	365.2	17.8	1.34925	0.41929	32	0.38396
12	402.6	17.66	1.28525	0.31352	28	0.37811
13	359	17.76	1.28525	0.44162	38	0.37811
14	403.4	17.75	1.28175	0.35332	24	0.37778
15	690	18.07	1.43675	0.36029	45	0.38632
16	427	18.27	1.3885	0.34289	19	0.38629
17	493.5	17.78	1.42425	0.3572	44	0.3855
18	352	18.13	1.35075	0.1617	13	0.37956
19	328	18.06	1.35075	0.3668	26	0.37956
20	384.7	17.88	1.331	0.33016	37	0.3855
21	384	17.98	1.44575	0.32152	57	0.3855

## Appendix C. Resolution or Source of Factor Names

**Factor Name**	**Resolution/Source**
Longitude (°E)	Field investigation
Latitude (°N)	Field investigation
Altitude (m)	Field investigation
Flower density	Field investigation
Flower species	Field investigation
Fractional vegetation cover (FVC)	500 m
Population density	1 km
Slope (°)	500 m
Annual average precipitation (mm)	1 km
Annual average temperature	1 km
Wind speed (m/s)	10 m
Annual average enhanced vegetation index	500 m
Annual maximum leaf area index	500 m
Relative humidity of soil	500 m
